# Association between Gout and Erectile Dysfunction: A Systematic Review and Meta-Analysis

**DOI:** 10.1371/journal.pone.0168784

**Published:** 2016-12-30

**Authors:** Xing-li Du, Lei Liu, Wen Song, Xiang Zhou, Zheng-tao Lv

**Affiliations:** 1 Nursing Management Department, Tongji Hospital, Tongji Medical College, Huazhong University of Science and Technology, Wuhan, Hubei, China; 2 Department of Urology, Union Hospital, Tongji Medical College, Huazhong University of Science and Technology, Wuhan, Hubei, China; 3 Department of Urology, Tongji Hospital, Tongji Medical College, Huazhong University of Science and Technology, Wuhan, Hubei, China; 4 Department of Internal Medicine V, University Hospital of Heidelberg, University of Heidelberg, Heidelberg, Germany; 5 Department of Orthopedics, Tongji Hospital, Tongji Medical College, Huazhong University of Science and Technology, Wuhan, Hubei, China; University of Sydney, AUSTRALIA

## Abstract

**Background:**

The aim of this systematic review and meta-analysis was to assess the possible association between gout and erectile dysfunction (ED).

**Methods:**

Studies were identified by extensively searching EMBASE, Pubmed, CENTRAL and ISI Web of Science. Four electronic databases were searched from their inception date to the latest issue (March 2016), without language restriction. Each reviewer screened articles independently and was blinded to the findings of the other reviewer. Data was extracted in adherence to the predetermined data collection form and meta-analysis was conducted via RevMan 5.3.

**Results:**

Five studies involving 56465 patients (mean age: 49.11 years) with gout and 155636 non-gout subjects (mean age: 48.76 years) were selected. The combination of unadjusted odds ratio (OR) showed that patients with gout were 1.44 times more likely to be diagnosed with ED when compared with control (95% confidence interval (95%CI) 1.20, 1.72). After adjustment for age and comorbidities, the heightened risk to develop ED was still present (1.18, 95%CI 1.02, 1.38). Subgroup-analysis by age showed statistically significant association of gout and ED in all age groups. However, evidence supporting a causal effect of gout on ED was insufficient.

**Conclusion:**

The findings of this review indicated a positive association of gout and ED, but this work is hampered by the heterogeneity among included studies, to some extent. Future studies with larger community-based homogeneous population and randomized controlled trials aimed to evaluate the effect of gout treatment on ED associated outcomes are needed at this point.

## Introduction

Gout is the most prevalent inflammatory arthritis in developed countries, especially in elderly men. It is an inflammatory arthritis caused by deposition of monosodium uric acid crystals in the joints and surrounding tissues (tophi) as a result of excess uric acid burden [1]. Gout has a relapsing and remitting clinical course with intermittent episodes of acute crystal-associated inflammatory arthritis or bursitis. However, patients with gout are not fully symptom-free between acute attacks, their quality of life is impaired during intercritical periods, as well as during flares probably because of low-grade inflammation associated with tissue deposits of urate in addition to associated comorbidities such as obesity, diabetes mellitus, hypertension, hyperlipidaemia and chronic renal disease [2–4].

Erectile dysfunction (ED) is defined by the National Institutes of Health as the inability to attain or maintain an erection sufficient for satisfactory sexual performance [5]. The estimated prevalence of ED in different countries is affected by the way the information is collected, the way the population is selected and sampled, and the way ED is defined [6]. Rosen and colleagues reported an overall prevalence of ED was 16% on a worldwide basis including the United States, Brazil, Mexico and five European countries [7]. The risk of ED is multifactorial, including age, smoking, diabetes, heart disease, depression and hypertension [8]. Aside from age and comorbidities, relational factors [9], psychiatric symptoms [10] might contribute to the pathogenesis of ED. Similarly, hypogonadism represents another important pathogenetic factors for ED [11].

Characterized by hyperuricemia, gout is a kind of polygenic disease which results from disorder of purine metabolism and/or impaired renal excretion of uric acid [12]. Uric acid has become an interesting potential risk factor for ED, as it is strongly linked with endothelial dysfunction [13–15], microvascular disease [16] and hypertension [17, 18]. The comorbidities associated with gout are closely connected with similar risk factors of vascular diseases [19]. Vitamin D deficiency might also explain the possible link between ED and gout [20]. In addition to this, gout per se is a source of stress, which would lead to the development of ED. Considering the fact that hyperuricemia can induce endothelial dysfunction, oxidative stress, inflammation, and microvascular disease, we consequently assume that there is a close connection between gout and ED. The possible relationship between gout and ED is still under investigation and the well-established information is very limited. To our knowledge, there is no published meta-analysis evaluating the association between gout and ED. Therefore, the aim of this review is to assess whether there is a relationship between ED and gout, and to quantify such associations using meta-analysis.

## Material and Methods

The protocol of this systematic review is registered with PROSPERO (registration number: CRD42016036638). This systematic review was conducted in accordance to the proposal for reporting Meta-analysis Of Observational Studies in Epidemiology (MOOSE) [21](S1 File). Our present study aims to determine whether men with gout have a higher risk of developing ED than men without gout.

### Search strategy

A systematic literature search of PubMed, EMBASE, the Cochrane Library and ISI Web of Science was conducted. All the above databases were searched from their inception dates up to the latest issue (March 2016). No language restriction was imposed. Medical subject Headings (MeSH) and free text words were used based on the specifications of each database. The following search strategy was used for the literature search in Pubmed, Cochrane Library and ISI Web of Science: ("Erectile Dysfunction"[Mesh] or Impotence or erectile dysfunction or male sexual impotence) and ("Gout"[Mesh] or Gout or gouts). In addition, the bibliographies of relevant systematic reviews and clinical guidelines were manually searched. The reference section for each study was also searched.

### Inclusion and exclusion criteria

To be considered for inclusion, the included studies were required to be observational studies (cross-sectional, cohort study, case-control, or epidemiological studies) investigating the association between gout and the risk of ED. The diagnosis of gout should be according to well-established criteria or based on a clinical diagnosis made by a clinical physicians. The primary outcome measures included unadjusted odds ratio (OR) or adjusted OR, these data should be provided in the original article or they could be calculated based upon the raw data. In case that data was not available in the original article, the corresponding author was connected via e-mail at least three times for the primary data. If the corresponding authors did not response after three e-mails, this article would not be used for quantitative synthesis. Unpublished articles were also included in our systematic review. Case reports, case series, book chapters, and editorials were excluded.

### Data extraction

Two investigators (XD and LL) screened each article independently and were blinded to the findings of the other reviewer. According to the predetermined inclusion criteria, two reviewers performed a strict screening to identify eligible articles independently. They extracted data independently from these selected articles using a standardized collection form, which included first author, country, year of the publication, study design, cohort sizes, demographic characteristics of participants in different groups, assessment of ED and gout, results and main findings.

Any disagreement between the two reviewers was resolved through discussion until a consensus was reached. The third review author (ZL) was consulted if a consensus could not be reached.

### Quality assessment

The Newcastle-Ottawa Scale (NOS) for the assessment of non-randomized studies was used to assess the risk of bias in case-control and cohort studies [22]. Agency for Healthcare Research and Quality (AHRQ) criteria was also utilized to assess the methodological quality of cross-sectional studies in this systematic review [23]. Two reviewers assessed the risk of bias among studies independently, the results were compared afterwards.

### Data Synthesis and Analysis

The meta-analysis and statistical analyses were performed by using RevMan 5.3 (Copenhagen: The Nordic Cochrane Centre, The Cochrane Collaboration, 2014). The association between gout and ED was estimated using adjusted OR and unadjusted OR, the upper and lower limits of the 95% confidence intervals (CI) were extracted from each study. The confounding factors considered included age, hypertension, diabetes and other comorbidities. Before the data of included studies was combined, heterogeneity among studies was estimated using a standard Chi-square test and the Higgins I^2^ test (P>0.1 and I^2^<50% indicate acceptable heterogeneity). We pooled data across studies using random effect models if statistical heterogeneity exist. When a low heterogeneity was detected, a random-effects model was also applied, since the validity of tests of heterogeneity could be limited with a small number of component studies.

In case of high heterogeneity, subgroup analysis was conducted based upon location, study design and age stratification. The ORs and the associated 95%CI in each age stratification were combined to eliminate the influence of age on the occurrence of ED. Considering age is the only confounding factor consistently presented in our selected studies, we did not perform subgroup analyses of other confounding factors. Sensitivity analysis was performed to determine the potential source of heterogeneity by removing each of the related studies one at a time and evaluating the resulting effect. Publication bias was assessed using Begg’s rank correlation test and Egger’s linear regression test via Stata version 12.0 (Stata Corp LP, USA).

## Results

### Literature search results

**Fig 1:**
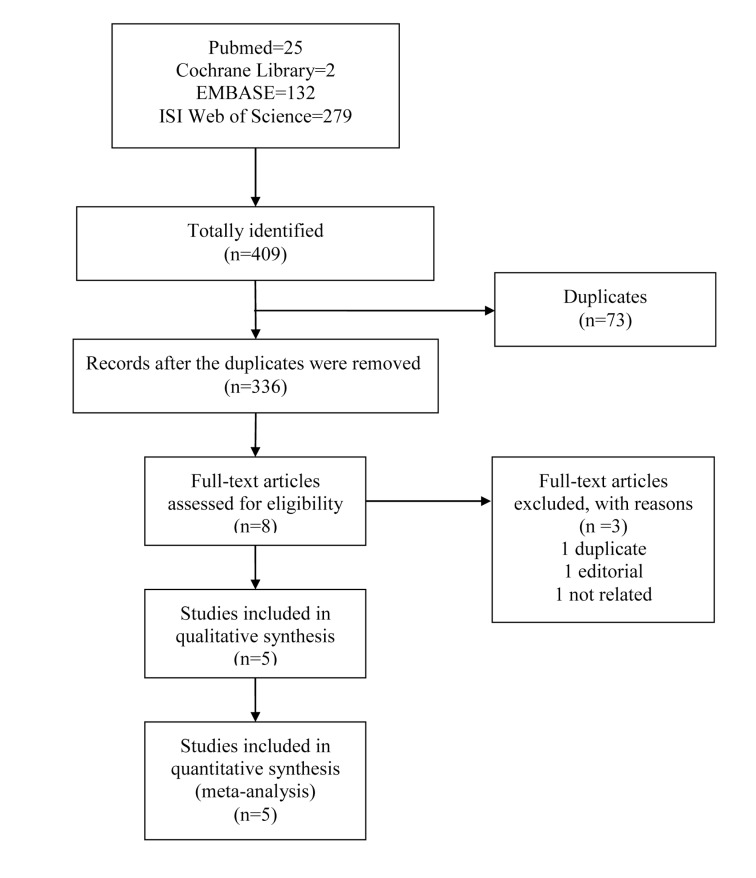
Flowchart of literature selection according to PRISMA guideline.

An initial search yielded 438 potential literature citations, including 25 records from Pubmed, 2 from Cochrane Library, 132 from EMBASE, and 279 from ISI Web of Science (Fig 1). 73 studies were deleted because they were duplicates. According to the predetermined selection criteria, 8 potentially relevant studies were selected and retrieved for a full-text assessment after exclusion at the title and the abstract stages. Of the remaining 8 articles, one was duplicate, one was editorial and one study only enrolled one patient with gout. Finally, a total of five studies [24–28] were deemed eligible for inclusion in this review.

### Study characteristics

Three published articles [24, 27, 28] and two conference publications [25, 26] were included in this work. The study design were either cross-sectional or cohort study. A total of 56465 patients with gout and 155636 non-gout subjects were included, the sample sizes ranged from 201 to 105794. Each study was conducted in a single center and published between 2010 and 2015. Assessment of gout and ED were inconsistent among studies, detailed data was presented in Table 1.

**Table 1:** Characteristics of included studies.

Study	Population (ED/total)	Gout assessment	ED assessment	Adjustment for covariates	Follow-up	Results
• Chen, 2015 • Cohort study • Taiwan	• Goat: 186/19368, 47.2±12 years • non-goat: 558/77472, 47.2±12 years	• ICD-9-CM Code 274 • Diagnosed by physicians	• ICD-9-CM Code 607.84 • identified by the urologists or physicians of internal or family medicine based on the patient’s medical record, recent symptoms, scores of the SHIM questionnaire, physical examination, and laboratory studies	age, congestive heart disease, ischemic heart disease, hypertension, depression and chronic renal failure	from January 2002 to December 201	• AOR: 1.21 (1.03, 1.44) • COR: 1.35 (1.14, 1.59)
• Hsu, 2015 • cohort study • Taiwan	• Gout: 476/35265, 49.6±16.2 years; • non-gout: 634/70529, 49.1±16.5 years	• ICD-9-CM code 274	PED or OED (ICD-9-CM codes 302.72 and 607.84)	age and coronary artery disease, peripheral arterial disease, chronic kidney disease, hypertension, diabetes, hyperlipidemia, depression and anxiety	• gout: 7.44±3.20 years • non-gout: 7.68±3.09 years	• AOR: 1.21 (1.07, 1.37) • COR: 1.46 (1.29, 1.64)
• Maynard, 2010 • cohort study • USA	• Gout: 102/256, 68.7±11.3 years; • non-gout: 677/2349, 65.0 ±12.3 years	health professional diagnosis of gout	health professional diagnosis of ED	age, hypertension, obesity and diabetes	N.R.	• AOR: 1.15 (0.85, 1.53) • COR: 1.64 (1.25, 2.13)
• Roddy, 2012 • cross-sectional study • UK	• Gout: 116/1292, 59.9 years; • non-gout: 429/5168, 59.9 years	identified via Read codes	identified via Read codes	ischemic heart disease, hypertension, diabetes mellitus, and prescription of diuretics, anti-hypertensives, H2 antagonists and anti-depressants	N.R.	• AOR: 0.97 (0.78, 1.22) • COR: 1.09 (0.88, 1.35)
• Schlesinger, 2015 • cross-sectional study • USA	• Gout: 63/83, 56.67±14.29 years; • non-gout: 60/118, 53.52±13.70 years	Reassessed retrospectively by investigating the medical reports	SHIM score: absent (22–25), mild (17–21), mild to moderate (12–16), moderate (8–11), and severe (1–7)	age, depression, diabetes, fasting glucose, HTN, elevated cholesterol level, prostate disease, GFR, and heart disease	N.R.	• AOR: 2.92 (1.41, 6.06) • COR: 3.04 (1.64, 5.66)

ED: erectile dysfunction; OED: organic erectile dysfunction; PED: psychogenic erectile dysfunction; COR: crude odds ratio; AOR: adjusted odds ratio; 95%CI: 95% confidence interval; HTN: hypertension; GFR: glomerular filtration rate; N.R.: not reported.

### Risk of bias assessment

The NOS scale was used to assess the risk of bias in the case-control and cohort studies, which evaluates three elements: the selection of the study groups, the comparability of the groups, and the evaluation of the exposure using a Star system with nine being the maximum possible score. Studies were categorized into low (scored 8–9), medium (scored 6–7), and high risk of bias groups (scored ≤5). Two cohort studies [27, 28] gained nine stars (Table 2).

**Table 2:** Risk of bias assessment of cohort studies.

Item/Study	Chen et al., 2015	Hsu et al., 2015
Selection		
1) Representativeness of the exposed cohort	*	*
2) Selection of the non-exposed cohort	*	*
3) Ascertainment of exposure	*	*
4) Demonstration that outcome of interest was not present at start of study	*	*
Comparability		
1) Comparability of cohorts on the basis of the design or analysis	**	**
Outcome		
1) Assessment of outcome	*	*
2) Was follow-up long enough for outcomes to occur	*	*
3) Adequacy of follow up of cohorts	*	*
Total score	9/9	9/9

A study could be awarded a maximum of one star (*) for each item within the selection. A maximum of two stars (**) can be given for comparability and selection. The definition/explanation of each column of the Newcastle-Ottawa Scale is available from (http://www.ohri.ca/programs/clinical_epidemiology/oxford.asp.).

Risk of bias assessment of cross-sectional study [24] was performed according to AHRQ criteria (Table 3). Schlesinger et al. enrolled patients presenting to the Rheumatology clinic with any form of arthritis between August 26, 2010, and May 13, 2013. The representativeness of the sample was insufficient and the authors did not describe the response rate or the characteristics of the responders and the non-responders. Furthermore, the subjects in different outcome groups were not comparable based on the study design or analysis, confounding factors were not well controlled. We did not assess the risk of bias in two conference publications since the provided information was insufficient.

**Table 3:** Risk of bias assessment of cross-sectional studies.

Item/Study	Schlesinger et al., 2015		
	Yes	No	Unclear
1) Define the source of information (survey, record review)	√		
2) List inclusion and exclusion criteria for exposed and unexposed subjects (cases and controls) or refer to previous publications	√		
3) Indicate time period used for identifying patients	√		
4) Indicate whether or not subjects were consecutive if not population-based		√	
5) Indicate if evaluators of subjective components of study were masked to other aspects of the status of the participants	√		
6) Describe any assessments undertaken for quality assurance purposes (e.g., test/retest of primary outcome measurements)	√		
7) Explain any patient exclusions from analysis		√	
8) Describe how confounding was assessed and/or controlled	√		
9) If applicable, explain how missing data were handled in the analysis		√	
10) Summarize patient response rates and completeness of data collection		√	
11) Clarify what follow-up, if any, was expected and the percentage of patients for which incomplete data or follow-up was obtained		√	

The definition/explanation of each column of the Agency for Healthcare Research and Quality is available from (http://www.ahrq.gov/research/findings).

### Meta-analyses Results

**Fig 2:**
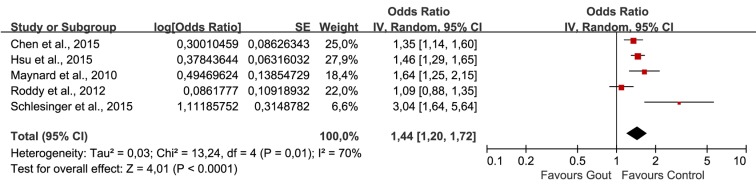
Forest plot of gout and ED: unadjusted OR.

**Fig 3:**
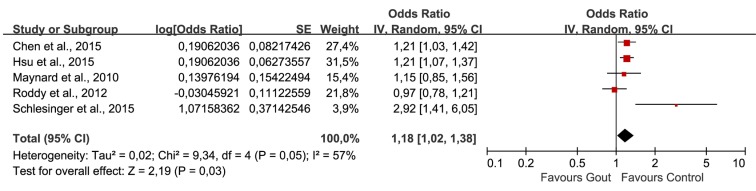
Forest plot of gout and ED: adjusted OR.

The combination of unadjusted ORs was shown in Fig 2. Heterogeneity test indicated obvious heterogeneity across studies (Tau^2^ = 0.03; Chi^2^ = 13.24; degree of freedom (df) = 4, P = 0.01; I^2^ = 70%), the random effect model was selected for statistical analysis. The pooled ORs showed that patients with gout were 1.44 times more likely to be diagnosed with ED than non-gout controls (Z = 4.01, P<0.0001; OR 1.44, 95%CI 1.20, 1.72). And the meta-analysis of adjusted OR using random effect model also revealed a statistically significant association of gout on ED (Z = 2.19, P = 0.03; OR 1.18, 95%CI 1.02, 1.38)(Fig 3). However, the heterogeneity among include studies was obvious (Tau^2^ = 0.02; Chi^2^ = 9.34; df = 4, P = 0.05; I^2^ = 57%).

### Subgroup analysis and sensitivity analysis

**Fig 4:**
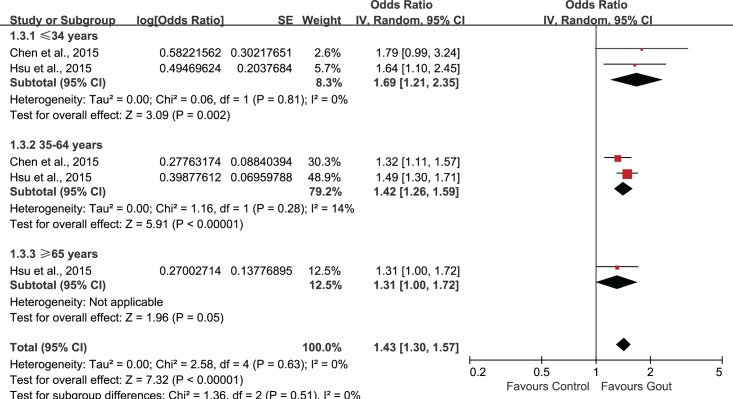
Subgroup-analysis based on age.

**Fig 5:**
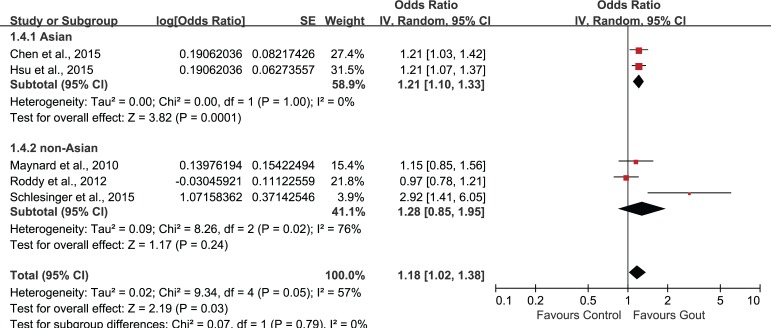
Subgroup-analysis based on study location (Asian/non-Asian).

**Fig 6:**
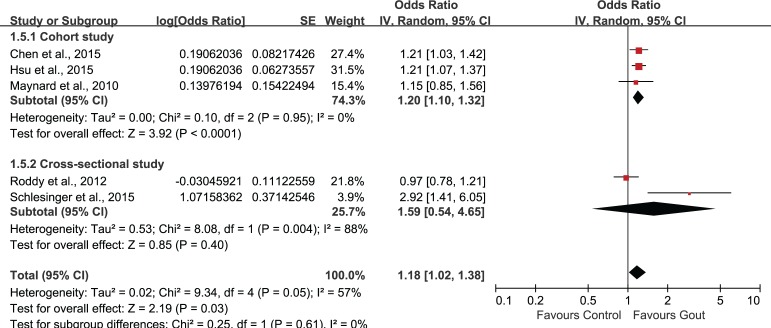
Subgroup-analysis based on study-design.

Subgroup analysis was performed according to age stratification (Fig 4). Statistically significant association of gout and ED could be observed in all age groups. The combined OR and associated 95%CI in ≤34, 35–64, ≥65 groups were 1.69 (1.21, 2.35), 1.42 (1.28, 1.58) and 1.31 (1.00, 1.72), respectively. Subgroup-analysis by study location (Asian/non-Asian) revealed a statistically significant correlation between gout and ED (OR 1.21, 95%CI 1.10, 1.33) in Asian countries, but this association was not significant in non-Asian countries (OR 1.28, 95%CI 0.85, 1.95)(Fig 5). In the subgroup-analysis by study-design, the combination of results from cohort studies showed statistically significant association between gout and ED (OR 1.20, 95%CI 1.10, 1.32), but the pooled data from cross-sectional studies suggested no significant association between gout and ED (OR 1.59, 95%CI 0.54, 4.65)(Fig 6).

**Fig 7:**
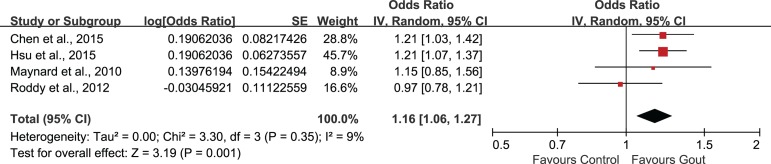
Sensitivity analysis.

Sensitivity analysis was performed by removing each of the related studies one at a time. After the exclusion of study conducted by Schlesinger et al. [24], the heterogeneity turned out to be acceptable (Tau^2^ = 0.00; Chi^2^ = 3.30; df = 3, P = 0.35; I^2^ = 9%). Thus, this study was deemed to be the main source of heterogeneity. The combination of the remaining four studies also indicated a statistically significant association between gout and ED (Z = 3.19; OR 1.16, 95%CI 1.06, 1.27; P = 0.001)(Fig 7).

### Publication bias

The Begg’s test (unadjusted OR: z = 0.73, P = 0.462; adjusted OR: z = -0.24, P = 1.00) and Egger’s test (unadjusted OR: t = 0.86, P = 0.451; adjusted OR: t = 0.78, P = 0.493) indicated no significant publication bias in the association between gout and ED.

## Discussion

To the best of our knowledge, this is the first systematic review to assess the correlation between gout and ED. The findings of our current study indicated a statistically significant risk of ED associated with gout, and this association existed in different age groups, even after adjustment of confounding covariates. The methodological quality of included studies was generally high, except one study was judged to medium risk of bias.

There are multiple factors involved in the pathogenesis of ED, including endothelial dysfunction, microvascular disease, alterations in testosterone levels, and psychological factors. The observation that uric acid may have a role in driving endothelial dysfunction and microvascular disease provides a potential causal link between uric acid and ED [29, 30]. Salem et al. reported that hyperuricemia could be regarded as an independent risk factor for ED in addition to the established ones [31]. ED is a common disease characterized by endothelial dysfunction, and uric acid itself causes endothelial dysfunction via decreased nitric oxide production and decreased vasodilator response to acetylcholine [32, 33]. Experimental studies also showed that uric acid can reduce endothelial nitric oxide bioavailability via multiple mechanisms, including scavenging by uric acid induced oxidative stress, the stimulation of arginase and direct scavenging [34–37].

Uric acid is not only associated with endothelial dysfunction, oxidative stress and inflammation, and is recently beginning to be recognized as a risk predictor for cardiovascular diseases (CVD) [38, 39]. Solak et al. concluded that for each change in 1mg/dl uric acid, there was a 31% increased risk for having ED; but when this correlation was adjusted for cardiovascular risk factors, the relationship with elevated uric acid levels was no longer significant [40]. Another study also reported a positive association between serum uric acid and increased risk of CVD, independent of renal function [41]. The Genetic Epidemiology Network of Arteriopathy study showed that serum uric acid was correlated with presence and severity of CVD after adjusting for age and gender, but not after adjustment for CVD risk factors [42]. Based on these findings, we are unable to conclude that uric acid is an independent risk factor for CVD. However, uric acid might have a contributory role to CVD as well as hypertension, insulin resistance, and renal function because of its pivotal role in causing endothelial dysfunction, oxidative stress, and inflammation.

ED not only shares many common risk factors with CVD but is also recognized as an incremental risk predictor for CVD. This is not surprising because CVD and ED share mutual risk factors such as diabetes mellitus, hypertension, advanced age, hypercholesterolemia, obesity, metabolic syndrome, certain medications such as antidepressants, and tobacco abuse [43–45]. Would treatment of hyperuricemia improve ED? Now-existing evidence seems to be insufficient. Experimental evidence using rat model showed that endothelial dysfunction could be reversed by normalizing the serum uric acid [46]. Others studies also reported that lowering serum uric acid with xanthine oxidase inhibitors can improve endothelial dysfunction [47–49]. An observational study conducted by Chen et al. indicated that receiving urate-lowering therapy (ULT) for more than 90 days may decrease the risk of ED development for patients with gout who had no comorbidity. However, compared with non-gout with no comorbidity, the patients with gout without comorbidity receiving fewer than 90 days of ULT remained at a greater risk of ED. These aforementioned studies are either experimental or observational, definite conclusion regarding the potential effect of ULT on ED associated outcomes could not be drawn. Thus, to determine the exact role of uric acid in ED, and subsequent CVD, future prospective randomized controlled trials are encouraged to explore whether a reduction in serum uric acid can prevent the development of ED and consequently CVD.

In the combination of adjusted ORs in our meta-analysis, Roddy et al. [25] as well as Maynard et al. [26] reported no significant difference between gout subjects and non-gout. The Campaign Against Cancer and Heart Disease study reported in abstract form that, ED was significantly more common among participants with gout as compared with participants without gout (39.8% vs 28.8%, p < 0.001). There was a statistically significant association between gout and ED, even after adjustment for age. However, this association was not observed after adjustment for hypertension, obesity, and diabetes. Roddy et al. reported that 116 (9.0%) men with gout consulted with ED compared to 429 (8.3%) of controls (adjusted OR 0.97; 95%CI 0.78, 1.22). We assume that relative advanced age of participants might dilute the influence of gout on ED, as advanced age is positive associated with development of ED. Unfortunately, detailed information about the experimental procedures could not be reached because two aforementioned studies are both published in abstract form. Except for advanced age, other comorbidities were also recognized to be factors associated with the odds of getting ED. Chen et al. reported that the incidence rate of ED for patients who had no gout but had 1 or more comorbidities was much higher than those who had gout but without comorbidities [28]. All the included studies reported lower adjusted OR than crude OR, suggesting that age and comorbidities could be risk factors of developing ED.

According to the result of sensitivity-analysis, the study conducted by Schlesinger et al. was excluded [24]. The results of meta-analysis was compared prior and after this study was excluded. The pooled data of the other four studies also indicated a statistically heightened risk of ED in patients with gout (1.16, 95%CI 1.07, 1.27). The dispersion of effect size among included studies was inconsistent. Schlesinger and colleagues reported a significantly higher OR (2.92, 95%CI 1.41, 6.05) than the other four studies included (1.16, 95%CI 1.07, 1.27). In this study, the enrolled subjects were adult men presenting to the Rheumatology clinic with any form of arthritis. Previous studies have shown that rheumatoid arthritis and systematic sclerosis were associated with an increased risk of ED [50, 51]. As Schlesinger and coworkers stated in their study, “Patients attending clinic more frequently were more likely to be included”. The reason for the stronger association detected by Schlesinger et al. may stem from a selection bias. Patients attending clinic more frequently would be more likely to pay attention to their health condition. On the other hand, the sample size of this cohort study was relatively small when compared with other included studies in our current review.

There are several limitations in our study. Firstly, two of our included studies were published in abstract form, making it impossible for us to achieve further information about the study design and experimental process. Secondly, few ED and gout associated clinical parameters were provided by our included studies: only one study reported serum uric acid levels of gout/non-gout subjects and only one study reported the severity of ED using SHIM. Thirdly, our included studies were lack of lifestyle information, such as physical activity levels and smoking status; it has been demonstrated that smoking is associated with increased ED, especially in young males with or without clinical cardiovascular disease, in various studies [52–54]. Lastly, the study-design of included studies were either cohort study or cross-sectional study, we quantified the association between gout and ED using meta-analysis, but the direction of causality was still unclear because cross-sectional studies have limitations for drawing causality once exposure is not temporally linked to outcome, so findings from this design may not represent the actual measurement of risk but come from reverse causality; to verify the hypothesis that gout has a causal effect on ED, randomized controlled trials to investigate whether ULT can prevent ED are needed.

## Conclusion

In conclusion, our systematic review and meta-analysis highlights a strong association between gout and ED, but the causal effect of gout on ED could not be confirmed due to limited evidence. Patients do not volunteer sexual complaints; therefore, it will be of benefit for the rheumatologists to consider gout as a risk factor of ED and inform patients with gout about the possible link with ED. Further scientific investigations are needed to explore the underlying mechanism of interaction between these two conditions.

## Take home messages

1. Our current review highlights a positive association between gout and erectile dysfunction.
2. Patients with gout are more likely to develop erectile dysfunction than non-gout subjects, and this association is consistent in different age groups.

## Supporting Information

S1 File PRISMA checklist. (DOC)
